# Supplementary material for: New Evidence on the Distribution of the Highly Endangered Natrix natrix cypriaca and Implications for Its Conservation
Source: Animals (Basel). 2021 Apr 9;11(4):1077. doi: 10.3390/ani11041077 (PMC8069274; doi:10.3390/ani11041077)
Supplement: Supplementary file 1 [file animals-11-01077-s001.pdf]

Natrix natrix - Rapit Assesment

Protocol for recording habitat suitability

Code:		Expert Opinion (Good/Medium/Bad):	
Date:		Time:	
Long (X):		Lat (Y):	
Recorded by:			

Natrix (Yes/No)	Rana frogs (Yes/No)	Water Crab (Yes/No)	Running waters (yes/no)	Small ponds (No, Yes 1-2, Yes few, Yes many)	Green plans on water (Yes / No)	River width (narrow/ openings / wide)	Slopes (Smooth, steep, Canyon)	Canopy (0-25 / 25-50 / 50-75 / 75-100) 0 = full sun /100 = no sun	Surrounding vegetation of bushes (Dense / sparce / absence)

Water pumps (Yes / No)	Mosquito spraying (Yes / No)	Hibernacula (Yes / No)	Nesting sites (Yes / No)	Busking sites (Yes / No)	Conservation Actions (if possible)	Other comments

Figure S1: Standardised protocol used during field survey for the rapid assessment of the riparian habitats and their suitability for *Natrix natrix cypriaca*.
